# Investigation of Interaction of Noble Metals (Cu, Ag, Au, Pt and Ir) with Nanosheets

**DOI:** 10.3390/mi12080906

**Published:** 2021-07-29

**Authors:** Mansoor H. Alshehri

**Affiliations:** Department of Mathematics, College of Science, King Saud University, Riyadh 11451, Saudi Arabia; mhalshehri@ksu.edu.sa

**Keywords:** noble metals, graphene, h-BN, mathematical modeling, Lennard–Jones potential

## Abstract

Two-dimensional nanomaterials, such as graphene and hexagonal boron nitride nanosheets, have attracted tremendous interest in the research community and as a starting point for the development of nanotechnology. Using classical applied mathematical modeling, we derive explicit analytical expressions to determine the binding energies of noble metals, including copper, silver, gold, platinum and iridium (Cu, Ag, Au, Pt and Ir) atoms, on graphene and hexagonal boron nitride nanosheets. We adopt the 6–12 Lennard–Jones potential function, together with the continuous approach, to determine the preferred minimum energy position of an offset metal atom above the surface of the graphene and hexagonal boron nitride nanosheets. The main results of this study are analytical expressions of the interaction energies, which we then utilize to report the mechanism of adsorption of the metal atoms on graphene and hexagonal boron nitride surfaces. The results show that the minimum binding energy occured when Cu, Ag, Au, Pt and Ir were set at perpendicular distances in the region from 3.302 Å to 3.683 Å above the nanosheet surface, which correspond to adsorption energies in the region ranging from 0.842 to 2.978 (kcal/mol). Our results might assist in providing information on the interaction energies between the metal atoms and the two-dimensional nanomaterials.

## 1. Introduction

**Figure 1:**
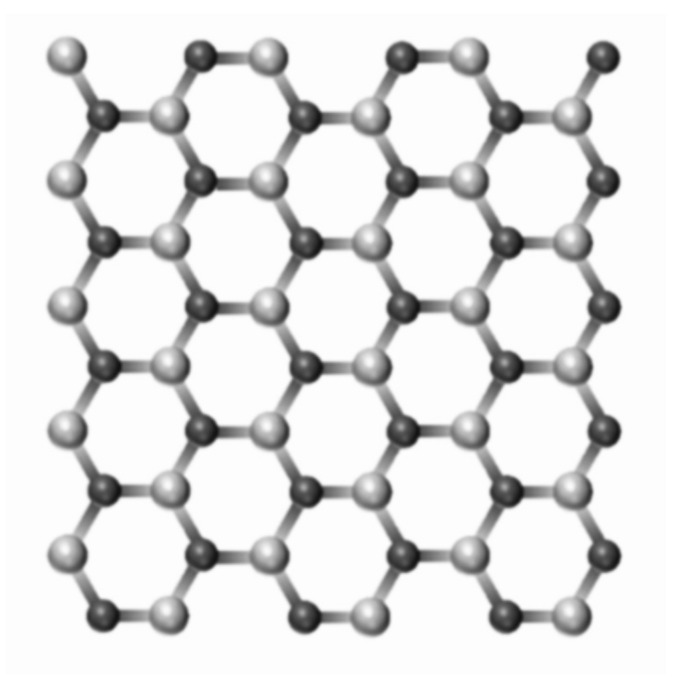
Two-dimensional sheet.

Recently, two-dimensional (2D) nanomaterials such as graphene (GRA) and hexagonal boron nitride (h-BN) have elicited increasing interest due to their unique structures and exceptional properties, and they have been significantly promoted due to their potential applications, including applications in the electronic, photonics, biomedical and bioassay fields [1,2,3]. Graphene nanosheets exhibited unusually high activity for oxidation reactions with metal clusters [4], which means that they attract attention as promising next-generation catalysts. Hexagonal boron nitride nanosheet (h-BN) is a two-dimensional nanomaterial, comprising a single layer of hexagonal rings of an equal proportion of boron (B) and nitrogen, as depicted in Figure 1. Compared with GRA, the h-BN nonosheet shows a high chemical stability due to its wide band-gap, and has a smooth surface without any charge traps, and it has a low dielectric constant and high-temperature stability, as well as a large ionicity. It is a good 2D insulator with a broad band gap [5,6]. The bond lengths in the C-C bonds in GRA and B-N bonds in h-BN are 1.42 Å and 1.45 Å, respectively [7]. Noble metals such as Cu, Ag, Au, Pt and Ir are metallic elements that serve as superior heterogeneous catalysts due to their resistance to oxidation, even at high temperatures; these metals can be used in new energy applications, medicine production and the petrochemical industry. The interactions of graphene and graphene-like materials with metals, including the noble metals, have been used for many potential applications, such as improving the chemical, thermal, mechanical, electrical and optical properties, and as biodevices, sensors, catalysis, energy storage and nanomagnetic materials [8,9,10,11]. A noble metals nanosheet may also be utilized as a conductive and stable support for an efficient single-atom catalyst (SAC). In addition, graphene and graphene-like materials have been used as templates for the synthesis of different noble-metal composites, providing a great performance in electrocatalytic applications, such as carbon dioxide (CO${}_{2}$) reduction and hydrogen evolution reaction [12]. Numerous computational and experiments were performed to study and evaluate the adsorption characteristics of graphene and graphene-like metals (see, for example, in [4,10,11,13,14,15,16,17,18,19]), but there are few works in this area that have used mathematical modeling, and new mathematical models are needed to secure the full import of nanotechnology into this area. Therefore, we used mathematical modeling techniques that are necessary to formulate explicit analytical criteria and ideal model behaviour to complement the efforts of experimentalists. The interactions between various nanostructured materials have been widely determined using mathematical modeling [20,21,22,23,24]. The standard method for this modeling is to employ the Lennard–Jones potential together with the continuous approximation; which is assumed that the atoms are uniformly distributed over the surface of the molecules [20]. As the dominant forces present within the interaction energies are van der Waals forces, the use of the Lennard–Jones potential is justified, and this approach enables the potential energy between the two interacting molecules to be obtained as a double integral over the two surfaces. This integral may be determined in terms of special functions, which is useful for the rapid evaluation of the numerical results for predictive purposes, and the accuracy of the results basically depends on the surface approximation [20]. In this work, mathematical modeling was used to adopt continuous approximation, together with the Lennard–Jones potential function, to study the adsorption of noble metals on GRA and h-BN nanosheets.

## 2. Method

**Figure 2:**
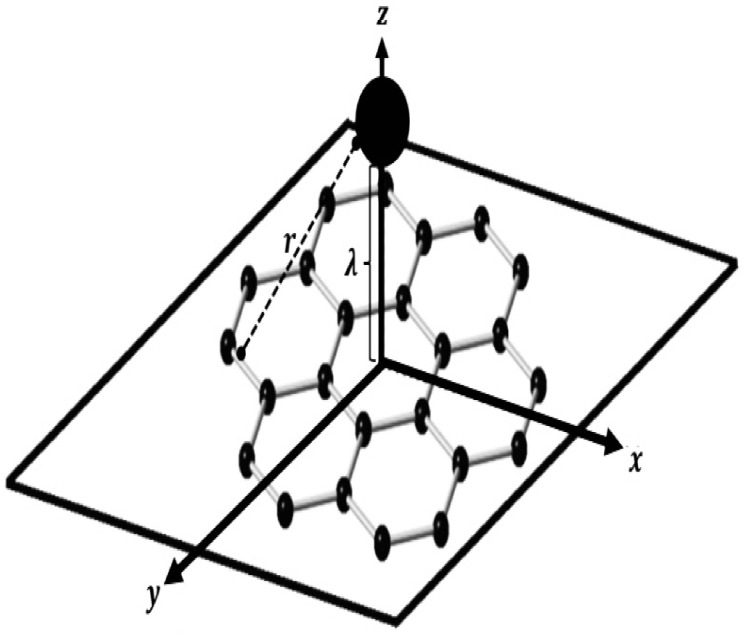
A sketch of the interaction between metal atoms and nanosheet.

We calculate the total interaction energies of Cu, Ag, Au, Pt and Ir atoms on the top position on graphene and hexagonal boron nitride nanosheets, as shown in Figure 2. The binding energies between atoms and nanosheets were determined using the 6–12 Lennard–Jones potential function, along with continuous approximation. The Lennard–Jones potential interaction energy between a pair of atoms separated by a distance *r* can be expressed as

$$\begin{array}{c} \Phi \left(r\right)=-\frac{A}{r^{6}}+\frac{B}{r^{12}}, \end{array}$$

where $A=4\varepsilon \sigma^{6}$ and $B=4\varepsilon \sigma^{12}$ denote the attractive and repulsive constants, respectively, calculated using the empirical mixing laws [25,26], and are listed in Table 1. Here, σ and ε are the van der Waals diameter and well depth, respectively, taken from Rappi et al. [27].

The non-bonded interactions between pair of atom, *n* and *m* are given by

(1) $$\begin{array}{c} E=\sum_{n}\sum_{m}\Phi \left(r_{nm}\right). \end{array}$$

Here, we use the 6–12 Lennard–Jones potential along with continuous approximation, thereby yielding the following equation:

(2) $$\begin{array}{c} E=h_{1}h_{2}\int_{M_{1}}\int_{M_{2}}\Phi \left(r\right)\;dM_{1}\;dM_{2}, \end{array}$$

where $h_{1}$ and $h_{2}$ are the mean atomic surface densities of atoms of the two interacting molecules. With reference to Figure 2, the nanosheets are assumed to lie flat on the xy–plane which is supposed an infinite plane, so, because of the large number of atoms of the 2D-nanomaterials (GRA and h-BN sheets), they can be modeled utilizing the continuous approximation. Equation (2) is presented the continuous approach, which is based on pair-wise summation of the interactions between molecules [28], this approach is assumed that the atoms are uniformly distributed over entire surfaces of the molecules. Herein, to model the the interactions between single atoms and molecules, such as nanosheets, an alternative hybrid discrete-continuous approach can be used. The said hybrid model, governed by elements of Equations (1) and (2), can be expressed as

(3) $$\begin{array}{c} E=\sum_{k}h\int \Phi \left(rk\right)\;dM, \end{array}$$

where Φ(rk) denotes the potential function; *r* denotes the distance between the atom and surface of the sheet, which can be modeled as the summation over each of its atoms and a typical surface element dM on the continuously modeled molecule. Additionally, *h* denotes the surface density of atoms on a sheet, which can be determined using the relation $h=4\sqrt{3}/9\varrho^{2}$, where ϱ is the length of one carbon–carbon or boron–nitride bonds. Thus, the atomic surface densities of graphene and boron-nitride nanosheets are $h_{g}=0.382$ and $h_{bn}=0.366$ Å${}^{-2}$, respectively. In addition, the approximate Lennard–Jones attractive and repulsive constants used in this study are listed in Table 1. Using the Cartesian coordinate system, a typical point on a nanosheet surface has the coordinates (x,y,0). With reference to Figure 2, the atoms are assumed to be located above the sheet surface with coordinates (0,0,λ), where λ is the perpendicular spacing between the atom and sheet plane. Therefore, the distance *r* between the atom and nanosheet surface can be obtained as

$$\begin{array}{c} r^{2}=x^{2}+y^{2}+\lambda^{2}. \end{array}$$

Thus, the interaction energies between the atoms and the nanosheet can be determined by performing a surface integral of the Lennard–Jones potential over the plane, namely,

$$\begin{array}{c} E=h_{SH}\int_{S}\left(-\frac{A}{r^{6}}+\frac{B}{r^{12}}\right)\;dS=h_{SH}\left(-AG_{3}+BG_{6}\right), \end{array}$$

where $h_{SH}$ $\left(h_{SH}\in \left\{h_{g},h_{bn}\right\}\right)$ is the mean surface density of atoms on the nanosheet. We begin by defining the integral $G_{n}$ (n=3,6) as

$$\begin{array}{c} G_{n}=\int_{-\infty }^{\infty }\int_{-\infty }^{\infty }\frac{1}{{\left(x^{2}+y^{2}+\lambda^{2}\right)}^{n}}\;dx\;dy=\int_{-\infty }^{\infty }\int_{-\infty }^{\infty }{\left(x^{2}+y^{2}+\lambda^{2}\right)}^{-n}\;dx\;dy. \end{array}$$

By making the substitution $y=\sqrt{x^{2}+\lambda^{2}}\tan\alpha$, thus integral $G_{n}$ becomes

$$\begin{array}{ccc} G_{n} & = & \int_{-\pi /2}^{\pi /2}\int_{-\infty }^{\infty }{\left[\left(x^{2}+\lambda^{2}\right)\tan^{2}\alpha +x^{2}+\lambda^{2}\right]}^{-n}\sqrt{x^{2}+\lambda^{2}}\sec^{2}\alpha \;dxd\alpha \\ & = & \int_{-\pi /2}^{\pi /2}\int_{-\infty }^{\infty }{\left(x^{2}+\lambda^{2}\right)}^{1/2-n}{\left(1+\tan^{2}\alpha \right)}^{-n}\sec^{2}\alpha \;dxd\alpha . \end{array}$$

Using $\sec^{2}\alpha =1+\tan^{2}\alpha$, the integral $G_{n}$ is given by

$$\begin{array}{c} E_{n}=\int_{-\pi /2}^{\pi /2}\int_{-\infty }^{\infty }{\left(x^{2}+\lambda^{2}\right)}^{1/2-n}\sec^{-(2n-2)}\alpha \;dxd\alpha , \end{array}$$

and from $\sec^{-(2n-2)}\alpha =\cos^{\left(2n-2\right)}\alpha$, $G_{n}$ becomes

$$\begin{array}{ccc} G_{n} & = & \int_{-\pi /2}^{\pi /2}\cos^{2n-2}\alpha \;d\alpha \int_{-\infty }^{\infty }{\left(x^{2}+\lambda^{2}\right)}^{1/2-n}\;dx\\ & = & 2\int_{0}^{\pi /2}\cos^{2n-2}\alpha \;d\alpha \int_{-\infty }^{\infty }{\left(x^{2}+\lambda^{2}\right)}^{1/2-n}\;dx, \end{array}$$

where the first integral can be evaluated using

$$\begin{array}{c} \int_{0}^{\pi /2}\sin^{\beta }\theta \cos^{\omega }\theta \;d\theta =\frac{1}{2}B\left(\frac{\beta +1}{2},\frac{\omega +1}{2}\right). \end{array}$$

Thus, the integral $G_{n}$ becomes

$$\begin{array}{ccc} G_{n} & = & B\left(n-1/2,1/2\right)\int_{-\infty }^{\infty }{\left(x^{2}+\lambda^{2}\right)}^{1/2-n}\;dx, \end{array}$$

where $B\left(\chi ,\kappa \right)$ is the beta function, and it may evaluate using

$$\begin{array}{c} B\left(\chi ,\kappa \right)=\frac{\Gamma (\chi )\Gamma (\kappa )}{\Gamma (\chi +\kappa )}=\frac{(\chi -1)!(\kappa -1)!}{(\chi +\kappa -1)!}, \end{array}$$

where a! is the factorial (a). On making a further substitution of x=λtanϕ, the integral $G_{n}$ becomes

$$\begin{array}{ccc} G_{n} & = & \lambda^{2-2n}B\left(n-1/2,1/2\right)B\left(n-1,1/2\right)\\ & = & \frac{\pi }{\left(n-1\right)\lambda^{2n-2}}. \end{array}$$

Thus, the total binding energy of the metal atoms on graphene and hexagonal boron nitride nanosheets is given by

(4) $$\begin{array}{c} E=h_{SH}\left(\frac{-\pi A}{2\lambda^{4}}+\frac{\pi B}{5\lambda^{10}}\right). \end{array}$$

## 3. Numerical Results

**Figure 3:**
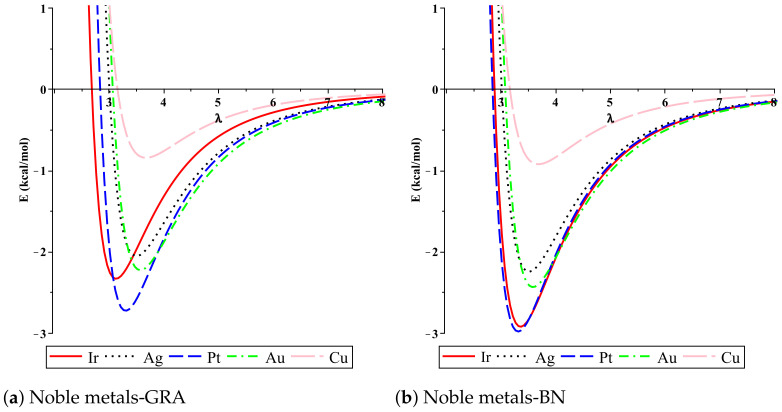
Interactions between noble metals and nanosheets with respect to the perpendicular distance λ.

**Figure 4:**
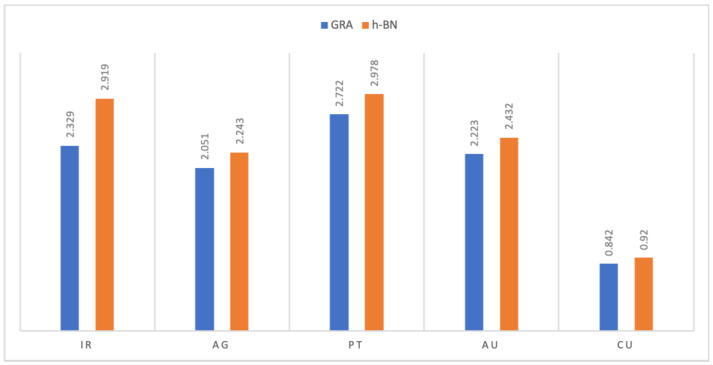
Comparison of the binding energies *E* (kcal/mol) of noble metal atoms on GRA and h-BN.

In order to find the numerical solutions to Equation (4) and evaluate the binding energies of metal atoms on graphene and hexagonal boron nitride nanosheets, we use the physical parameters involved in the model, together with the algebraic computer package MAPLE. We plot the binding energies for the five atoms of metal, including Cu, Ag, Au, Pt and Ir, situated above the nanosheet surface. In Figure 3, we show, for each atom, the relationship between the binding energy *E* and the equilibrium distance λ. Our results establish that the binding energy of the metal atoms on both graphene and hexagonal boron nitride nanosheets in the region from 0.842 to 2.978 (kcal/mol), in the order of Pt > Ir > Au > Ag > Cu, which is dependent on the the physical parameters of the atom and the sheet. We note that the values of the interaction energies between the atoms and GRA and h-BN sheets are sensitive to the Lennard–Jones parameters (A and B), which are calculated using the values of the van der Waals diameter and the well depth, that can be shown from the difference of the results. We also show graphically, in Figure 4, the comparison of the bind energy of each atoms on GRA and h-BN nanosheets; this comparison shows that the interactions of the atoms with h-BN are larger than the interactions of the atoms with GAR. Although the structure of the GRA and h-BN sheets are similar, (h-BN) possesses quite different properties: for example, the h-BN sheets may be more uniform in terms of electronic properties than teh GRA nanosheet, which might effect the interactions. Moreover, Table 2 shows the details of the main results of the binding energies and equilibrium distances λ of metal atoms with nanosheets. The results observe the optimal equilibrium distances of the metal atoms above both GRA and h-BN nanosheets, which are in the region from 3.302 Å to 3.683 Å. The results can be compared favorably with others, it can be seen that there is a quite good agreement in the case of GRA, while in the h-BN case there are differences, as shown in Table 3. We comment that, the discrepancy between our results in some cases from the others may be attributed to the physical parameters and the geometric structure which we have adopted here.

## 4. Summary

We investigated the behavior of the binding energies of noble metals, including Cu, Ag, Au, Pt and Ir atoms adsorption, on graphene and hexagonal boron nitride nanosheets by using the 6–12 Lennard–Jones potential function, together with the continuous approach. The numerical results for the binding energies of metal atoms on graphene and hexagonal boron nitride nanosheets are described very well using the algebraic computer package MAPLE. The results show that the binding energies of some of the five atoms on GRA and h-BN nanosheets can stronger than the others; furthermore, the binding energies of the five atoms on h-BN nanosheets are stronger than the binding energies on GRA, which might promote important applications in the area of molecular electronics. Moreover, the results can provide the fundamental information of the metal–two-dimensional nanomaterial junction, which might attract more attention for designs such as transistors and nano-devices using nanosheets. In addition, our method and results might assist in the study and development of the nanosheet–noble metal composites and their applications.

## Figures and Tables

**Table 1:** Approximate Lennard–Jones attractive and repulsive constants.

Noble Metals	GRA		h-BN	
	A (Å${}^{6}$ kcal/mol)	B (Å${}^{12}$ kcal/mol)	A (Å${}^{6}$ kcal/mol)	B (Å${}^{12}$ kcal/mol)
Ir	326.462341	304,333.744600	515.216085	735,744.470000
Ag	451.691459	829,617.425600	473.585871	885,229.408000
Pt	475.616149	617,040.328800	499.191704	659,780.286800
Au	531.686515	1,104,394.78400	557.260220	1,177,588.05000
Cu	225.042847	552,574.428400	235.755904	588,639.989600

**Table 2:** Main results of the binding energies *E* and equilibrium distances λ.

Noble Metals	GRA		h-BN	
	*E* (kcal/mol)	λ (Å)	*E* (kcal/mol)	λ (Å)
Ir	2.329	3.125	2.919	3.355
Ag	2.051	3.499	2.243	3.509
Pt	2.722	3.302	2.978	3.313
Au	2.223	3.572	2.432	3.582
Cu	0.842	3.672	0.920	3.683

**Table 3:** Comparison of the values of the equilibrium distances of this study and other studies.

Atom	Equilibrium Distances λ (Å)			
	GRA		h-BN	
	This Study	Other Studies	This Study	Other Studies
Cu	3.672	3.26 [29] and 2.5 [11]	3.683	2.80 [16] 1.14 and [14]
Au	3.572	3.31 [29] 3 and [11]	3.582	3 [30] and 1.58 [14]
Pt	3.302	3.30 [29]	3.313	2.4 [31] and 1.42 [14]
Ag	3.499	3.33 [29], 3.4 [11] and 3.7 [32]	3.509	1.66 [14]
Ir	3.125	2.37 [33]	3.355	1.34 [14]
